# Immunization with a 22-kDa outer membrane protein elicits protective immunity to multidrug-resistant *Acinetobacter baumannii*

**DOI:** 10.1038/srep20724

**Published:** 2016-02-08

**Authors:** Weiwei Huang, Yufeng Yao, Shijie Wang, Ye Xia, Xu Yang, Qiong Long, Wenjia Sun, Cunbao Liu, Yang Li, Xiaojie Chu, Hongmei Bai, Yueting Yao, Yanbing Ma

**Affiliations:** 1 Laboratory of Molecular Immunology, Institute of Medical Biology, Chinese Academy of Medical Sciences & Peking Union Medical College; Kunming, China 650118; 2 Yunnan Key Laboratory of Vaccine Research & Development on Severe Infectious Diseases; Kunming, China 650118; 3 Yunnan Engineering Research Center of Vaccine Research and Development on Severe Infectious Diseases, Kunming, China 650118

## Abstract

*A. baumannii* infections are becoming more and more serious health issues with rapid emerging of multidrug and extremely drug resistant strains, and therefore, there is an urgent need for the development of nonantibiotic-based intervention strategies. This study aimed at identifying whether an outer membrane protein with molecular weight of about 22 kDa (Omp22) holds the potentials to be an efficient vaccine candidate and combat *A. baumannii* infection. Omp22 which has a molecule length of 217 amino acids kept more than 95% conservation in totally 851 reported *A. baumannii* strains. Recombinant Omp22 efficiently elicited high titers of specific IgG in mice. Both active and passive immunizations of Omp22 increased the survival rates of mice, suppressed the bacterial burdens in the organs and peripheral blood, and reduced the levels of serum inflammatory cytokines and chemokines. Opsonophagocytosis assays showed *in vitro* that Omp22 antiserum had highly efficient bactericidal activities on clonally distinct clinical *A. baumannii* isolates, which were partly complements-dependent and opsonophagocytic killing effects. Additionally, administration with as high as 500 μg of Omp22 didn’t cause obvious pathological changes in mice. In conclusion, Omp22 is a novel conserved and probably safe antigen for developing effective vaccines or antisera to control *A. baumannii* infections.

*Acinetobacter baumannii* (*A. baumannii*) has recently emerged to be an important conditioned nosocomial pathogen that may cause pneumonia, septicemia, urinary tract infections, and meningitis^1^. It has been included in the Infectious Diseases Society of America (IDSA) hit list of the six most dangerous microbes^2^. The most problematic issues with *A. baumannii* are rapid emerging of multidrug and extremely drug resistant strains and the slow development of new antibiotics^3^,^4^,^5^,^6^. Therefore, there is an urgent need for the development of nonantibiotic-based intervention strategies to combat this pathogen^7^. Vaccine is one of the most effective intervention strategies for infection control, and functioning through approaches that differ from that of antibiotics, it is likely to circumvent complex multidrug-resistant mechanisms of *A. baumannii.* The immunogen candidates, reported previously to have provided potential immune protection against *A. baumannii* infection, include iron-regulated outer membrane proteins (IROMP)^8^, formalin-inactivated whole cells (IWCs)^9^, outer membrane complexes (OMCs)^10^, outer membrane vesicles (OMVs)^11^,^12^, biofilm-associated protein (Bap)^13^, poly-N-acetyl-β-(1–6)-glucosamine (PNAG)^14^, trimeric autotransporter protein (Ata)^15^, K1 capsular polysaccharide^16^, and outer membrane protein A (OmpA)^17^. However, of these candidates, IWCs, OMCs and OMVs have complex compositions and some of the identified subunit protein antigens such as OmpA have shown to be toxic^18^,^19^,^20^, which maybe cause safety concerns with their use and thus prevent their further development to be a clinically applicable vaccine. It is important for effectively fighting against *A. baumannii* infection to identify safer antigen candidates that hold the capability of eliciting protective immunity and providing cross-protection against varied clinical *A. baumannii* strains.

Previous studies have shown that immunization with OMVs provided strong immune protection against infections of not only homologous strains but also clonally distinct clinical isolates of *A. baumannii.* OMVs contain highly immunogenic outer membrane proteins, which may significantly contribute to eliciting protective immunity^12^. In this current study, we successfully identified an outer membrane protein (Omp22, with a molecular weight of 22.35 kDa) from OMVs as a potential vaccine candidate.

## Results

### Omp22 is highly conserved outer membrane protein in *A. baumannii*

**Figure 1:**
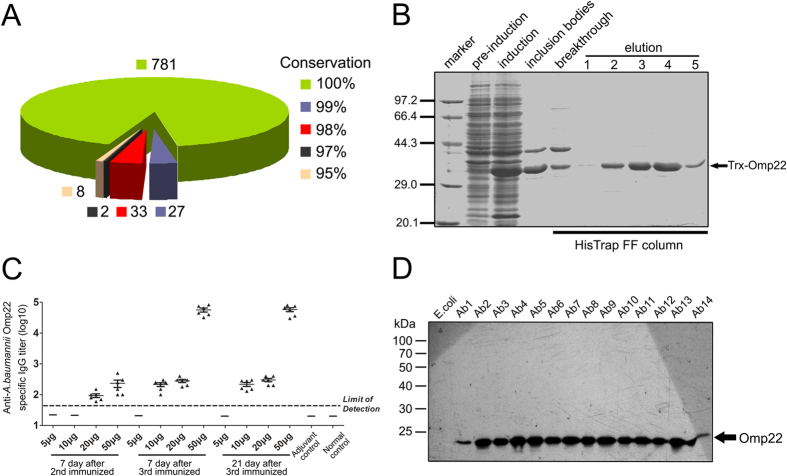
Conservation analyses, protein preparation, antibody induction, and expression levels in clinical isolates of Omp22. (**A**) Conservation analyses of Omp22 in reported *A. baumannii* strains using NCBI BLAST. The pie chart showed the strain numbers in conservation of 100%, 99%, 98%, 97% and 95% to sequence in this study, respectively. (**B**) SDS-PAGE analyses on Trx-Omp22 expression and purification. The 1–5 showed elution fractions from HisTrap FF column. (**C**) Titers of anti-*A. baumannii* IgG in mice immunized with different doses of Omp22 were detected by ELISA (n = 6 mice/group). Sera from mice receiving adjuvant only and normal mice were used as control. (**D**) Omp22 expression levels in clinical isolates Ab1-14 was measured with immune blotting. *E. coli* BL21 (DE3) cells were used as a control.

The DNA fragments encoding for Omp22 were amplified from ATCC 17978 and 14 clinical isolates. Alignment analyses of nucleotide sequences and amino acid sequences showed that the nucleotide sequences of Omp22 from all the strains had only one nucleotide mutation (T-A) at position 495; it was T in Ab1 and Ab3 isolated from the First Affiliated Hospital and Ab5, Ab8, Ab9, Ab10, Ab12, and Ab14 isolated from the Second Affiliated Hospital, while it was A in the other strains; the nucleotide change was a synonymous mutation for the amino acid sequence and maintained the related code translated into arginine (R) in all the strains. And thus, Omp22 protein is completely conserved among all the analyzed strains including ATCC strain and the 14 clinical isolates. Further, using NCBI BLAST, the amino acid sequence obtained in this current study was compared with previously reported *A. baumannii* Omp22 sequences in the data bank. Among the total 851 amino acid sequences, 781, 27, 33, 2, and 8 sequences showed correspondingly 100%, 99%, 98%, 97% and 95% conservation to our sequence (Fig. 1A). In addition, PubMed BLAST search of the human proteome using the ATCC 17978 Omp22 sequence revealed that only 10 sequences had homology with Omp22 (E values ranging 0.41 to 9.9), and the largest number of consecutive identical amino acids were less than six. The results showed that Omp22 was highly conserved across a broad array of clinical isolates of *A. baumannii* and shared almost negligible homology with human proteins.

### Specific IgG response is induced efficiently by purified recombinant Omp22 in mice

Recombinant Omp22 was expressed successfully in *E. coli* cells (Fig. 1B), as a fusion protein with thioredoxin ligated at N terminal. Majority of the expressed Trx-Omp22 presented as insoluble inclusion bodies. Subjected to a protein purification procedure consists of denaturation, refolding, and affinity chromatography, refolded recombinant Trx-Omp22 protein was prepared with a relatively high purity of more than 96% which was roughly analyzed by density scan for the stained bands on the SDS-PAGE gel using an Image lab software (Bio-Rad) (Fig. 1B).

Serum samples collected from immunized mice were used for detecting specific antibody response. The results showed that the doses of 20 μg and 50 μg elicited low titers of specific IgG at one week after the second immunization, and 50 μg produced high titers of the specific antibodies after the third immunization and maintained at least for three weeks; the doses of 10 μg and 20 μg induced low levels even after the third immunization; and mice received 5 μg dose didn’t show specific antibody response (Fig. 1C).

The Omp22 expression in clinical isolates Ab1 through 14 were analyzed by immune blotting using obtained antiserum from immunized mice to provide detection antibodies. The antiserum specifically reacted with all clinically isolates, showing a band with molecular weight around 22 kDa (Fig. 1D). The result indicated that Omp22 was expressed at a similar level in clinical isolates except for Ab1 strain, which showed a dramatically reduced Omp22 expression in comparison with the others. In contrast, there was no reactive bands showing for the *E,coli* BL21(DE3) sample, suggesting that antiserum of *A. baumanii* Omp22 didn’t recognize any protein in *E. coli* cells.

### Mice receiving Omp22 immunization shows an increased survival and reduced bacterial burdens in a sepsis model

**Figure 2:**
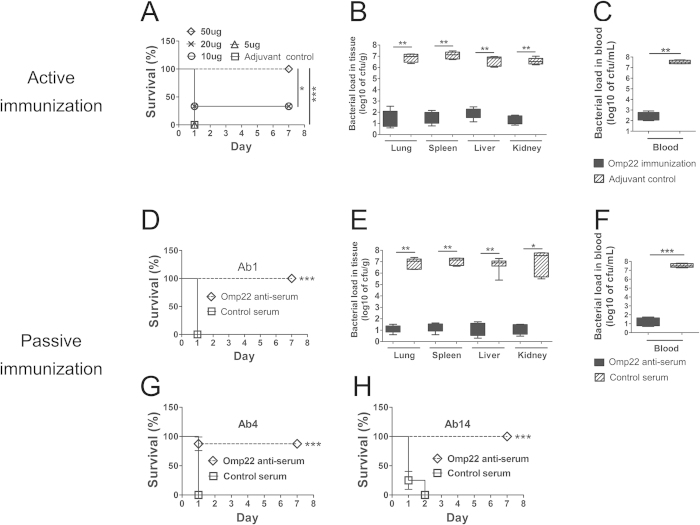
Immunization with Omp22 increases survival and reduces the bacterial burden of mice. (**A**) Active immunization with different doses of Omp22 protected mice from lethal challenge of clinical strain Ab1 in a sepsis model. The mice were monitored twice a day for 7 day. The mice receiving adjuvant only were used as control (n = 6 mice/group). Mice were challenged (i.p,) by Ab1 at day 49, which is 21 day after the last immunization. (**B**) Active immunization with 50 μg/mL of Omp22 significantly reduced bacterial burdens in main organs and (**C**) blood, determined at 12 h after Ab1 infection (n = 6 mice/group). (**D**) Passive immunization with antisera protected mice from lethal challenge of clinical strain Ab1 in a sepsis model. 100 μL antisera were injected into tail veins of mice 1 h before Ab1 challenge, and serum from mice receiving only adjuvant was used as a control (n = 6 mice/group). (**E**) Passive immunization with antisera significantly reduced bacterial burdens in main organs and (**F**) blood (n = 6 mice/group). *p < 0.05, **p < 0.01, ***p < 0.001. (**G**) and (**H**) Passive administration of Omp22 antisera also protected mice from lethal challenge of clonally distinct clinical isolates Ab4 and Ab11. ***P < 0.001, n = 8/group.

After the specific antibody responses were induced by immunization with recombinant Trx-Omp22, an intraperitoneal challenge of a clinical isolate Ab1 was performed. All the mice receiving only the adjuvant as control died within 24 h. The survival rates were 100%, 33%, 33%, and 0% for the mice receiving 50 μg, 20 μg, 10 μg, and 5 μg of Trx-Omp22, respectively. The results demonstrated that the protective effects found in the immunized mice were basically in an immunization dose–dependent manner (Fig. 2A). Together with the finding that high doses of Omp22 elicited high titers of specific antibodies, the results might indicate an important role of the specific antibodies in providing immune protection against *A. baumanii* infection. Bacterial burdens in major organs and blood were also detected to reflect protective effects of immunization with Trx-Omp22. Mice receiving 50 μg proteins showed significantly reduced bacterial burdens in the lung, spleen, liver, kidney and blood by approximately 10^4^–10^5^-folds, when compared with those in the mice receiving only adjuvant (Figs 2B,C). The results implied that systemic anti-*A. baumannii* antibodies produced by Omp22 immunization facilitated the clearance of *A. baumannii*.

In the study with passive immunization, administration of antiserum resulted in 100% survival of mice, while mice treated with the control serum, which was collected from mice receiving adjuvant only, all died within 24 h post infection (Fig. 2D). At 12 h after infection, the bacterial burdens in the lung, spleen, liver, and kidney tissues in mice receiving antiserum decreased by approximately 10^5^–10^6^ folds compared with those in mice receiving control serum (Fig. 2E); and, the bacterial burden in the peripheral blood also decreased by approximately 10^6^ folds (Fig. 2F). Passive immunization with Omp22 antisera also effectively protected mice from lethal challenges of clonally distinct clinical isolate Ab4 and Ab14 which showed higher Omp22 content than Ab1 (Figs 2G,H).

### Immunization with Omp22 reduces serum proinflammatory cytokine and chemokine levels

**Figure 3:**
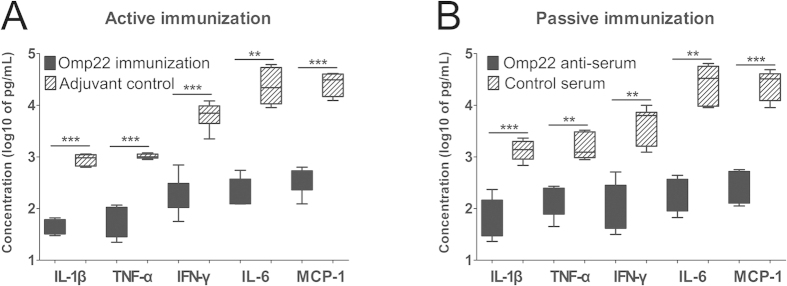
Immunization with Omp22 reduces serum proinflammatory cytokine and chemokine levels. (**A**) Active immunization. (**B**) Passive immunization. ELISAs were performed using the sera collected from mice receiving either Trx-Omp22 or adjuvant only in a sepsis model. (n = 6 mice/group). **P < 0.01, ***P < 0.001.

To determine whether or not immunization with recombinant Omp22 is able to reduce inflammatory responses induced by *A. baunmanii* infection, cytokine and chemokine levels in serum were measured at 12 h post infection. Levels of IL-1β, IL-6, TNF-α, IFN-γ and MCP-1 were significantly lower in both actively (Fig. 3A) and passively immunized mice (Fig. 3B) than in control mice, indicating immunization suppressed inflammatory cells accumulation and cytokine release, possibly due to quick clearance of bacteria mediated by specific antibodies.

### Antisera of Omp22 provide effective opsonophagocytic killing against clinical *A. baumannii* isolates

**Figure 4:**
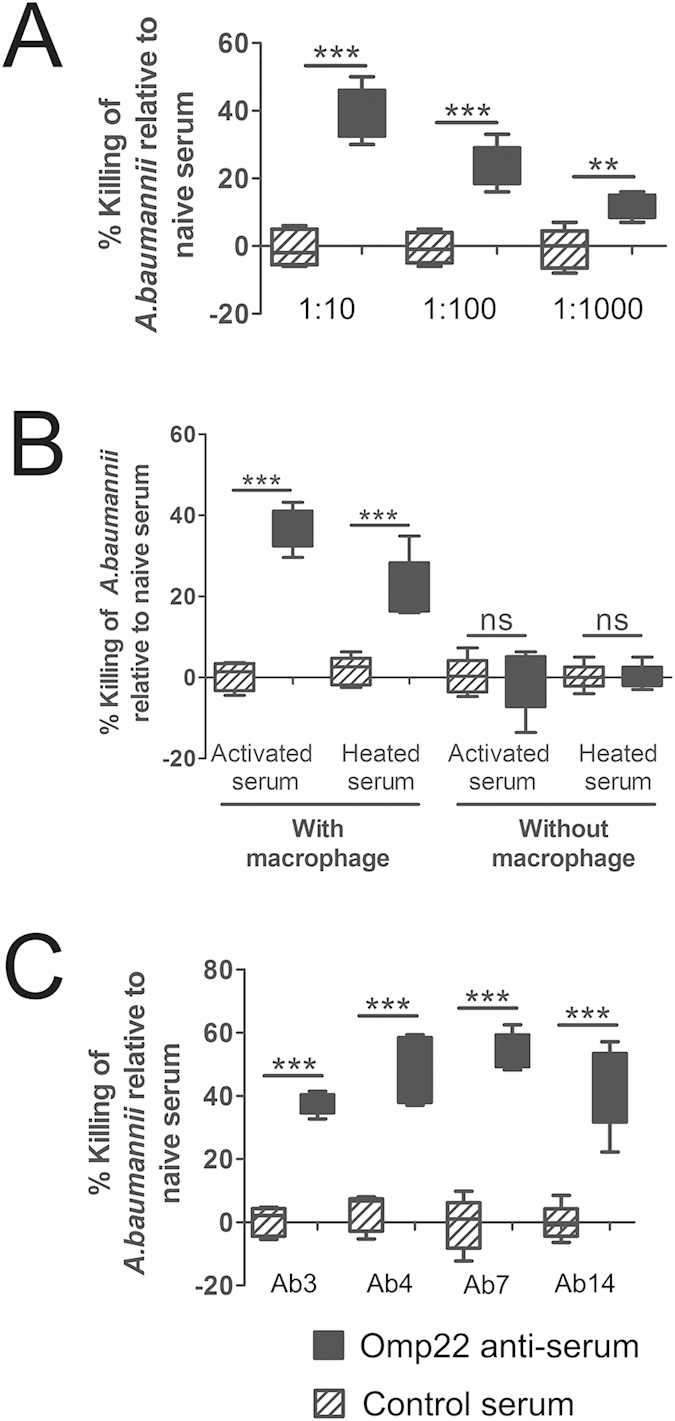
Antisera of Omp22 provide effective opsonophagocytic killing against clinical *A. baumannii* isolates. **A**) The bactericidal killing activities of different concentrations of Omp22 antiseram on clinical strain Ab1. The Omp22 antisera and adjuvant control serum were compared with naïve serum. The difference between the Omp22 antisera and the adjuvant controls were statistically analyzed. (**B**) The bactericidal killing mechanism is antiserum-mediated and partly complements-dependent opsonophagocytosis effects. The complement and opsonophagocytic assays were performed with serum (1:10) heated to inactivate the complement components and with macrophage RWA264.7 cells removed. (**C**) The Omp22 antisera against *A. baumannii* ATCC 17978 had the potential to opsonize against challenges with non-homologous strains. A 1:10 dilution of serum was used. Experiments were performed in quintuplicate. **P < 0.01, ***P < 0.001.

To elucidate the possible mechanism underlying protective effects of immunization targeting to Omp22, we evaluated opsonophagocytic effects of the antisera on clinical isolate Ab1. The results showed that the antisera presented 39.8% specific bactericidal activity at dilution of 1:10, 24% at 1:100, and 11.8% at 1:1000, as compared to the sera from naïve mice. The effect is in an antiserum concentration-dependent manner. There was no significantly different between the sera from mice receiving only adjuvant and naïve serum (Fig. 4A).

To investigate the roles of complement components in Omp22 antiserum-specific opsonophagocytic killing on *A. baumannii,* endogenous complements in the serum samples were heated to be inactivated. The complements-inactivated antisera showed a 22.2% killing efficiency at dilution of 1:10. Comparing with 36.6% killing rate of non-inactivated antiserum, the reduced 14.4% killing rate, which was equivalent to 39% of total killing rate, was attributed to complement components. Therefore, the bacterial killing effects mediated by antibodies were partly complement-dependent. There was no difference between inactivated and non-inactivated control sera (Fig. 4B)

Furthermore, macrophages were removed from the system with activated or heated serum to display the possible direct bactericidal effects of serum components such as antibodies and complements. There were no killing activities on bacteria shown with the removal of macrophages, indicating that the main mechanism was phagocytes-dependent opsonophagocytic killing and direct effects of serum components were limited (Fig. 4B).

Even though amino acid sequences of Omp22 in ATCC 17978 and 14 clinical isolates are identical, we still investigated the *in vitro* killing activity on different *A. baumannii* strains in case actual expression, distribution and exposure of Omp22 might vary and have significant influences on the opsonophagocytic killing effects. The results showed that the mean killing activities were 37.48% for Ab3, 47.66% for Ab4, 54.4% for Ab7, and 42.6% for Ab14, at antiserum dilution of 1:10 (Fig. 4C).

### Omp22 preparation presents mild suppressing effects on the growth of A549 and 293FT cells *in vitro*

**Figure 5:**
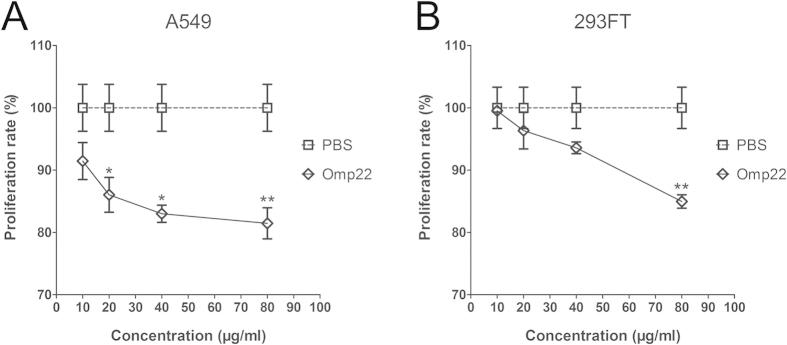
Omp22 preparation mildly suppresses the growth of 293FT and A549 cells *in vitro*. The possible cytotoxicity of Trx-Omp22 on A549 cells (**A**) and 293FT cells (**B**) was analyzed using an MTS cell proliferation assay. The cells were incubated with Trx-Omp22 at concentrations of 0, 10, 20, 40 or 80 μg/ml for 24 h. *P < 0.05, **P < 0.01, Experiments were performed in triplicate.

The functions of Omp22 in *A. baumannii* as well as its possible roles in interacting with cells of infected individuals have not been reported yet. An assay of cell proliferation was performed to roughly evaluate *in vitro* the possible toxicity of Omp22. The results showed that Omp22 only had a mild inhibitory effect on proliferation of the tumor cell line A549 and the normal cell line 293FT. Although suppressive effects began at the concentration of 20 μg/ml, A549 cells treated with a dose as high as 80 μg/ml of Omp22 for 24 h still maintained 81.45% viability (Fig. 5A); in 293FT cells, treatment with 80 μg/ml Omp22 decreased the cell viability to 84.96% while treatment with 10. 20, and 40 μg/ml of Omp22 maintained above 90% cell viability (Fig. 5B). The results may imply that the purified Omp22 preparation have only mild cytotoxity on mammalian cells and likely to be a safe vaccine candidate.

### Administration with high dose of Omp22 don’t cause obvious pathological changes in mice

**Figure 6:**
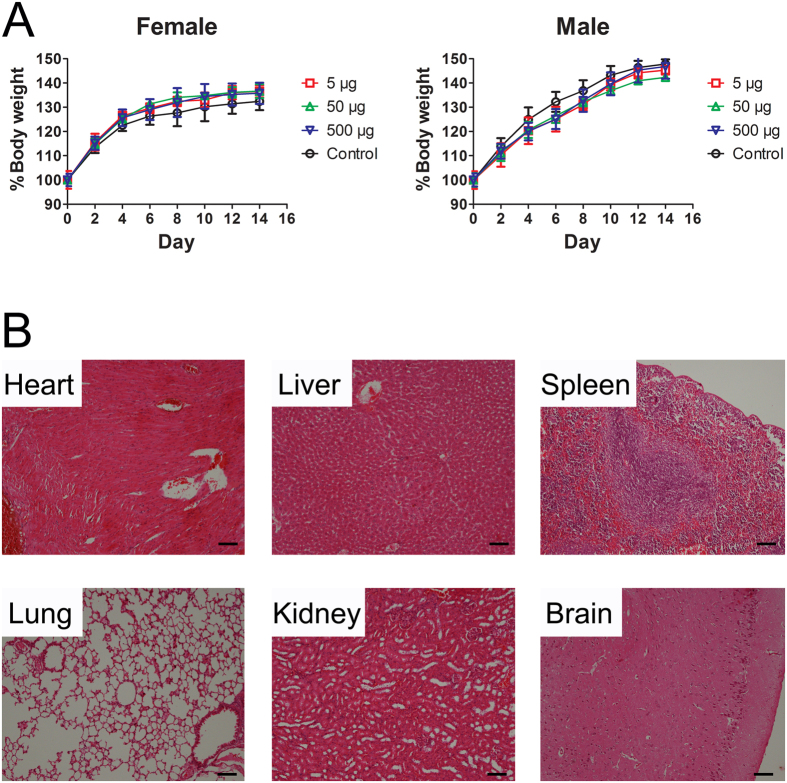
The acute toxicity test of Omp22 in mice. (**A**,**B**) Bodyweight changes of female and male ICR mice. Mice were given a single subcutaneous injection with either PBS or Omp22 at day 0. Data are expressed as mean ± SEM (n = 4/group). (**C**) Histology assessment. Tissue sections of brain, liver, spleen, heart, lung and kidney were stained with H&E, and the representative photomicrographs from mice receiving the highest dose (500 μg) of Omp22 were shown. Bar: 100 μm.

Mice were administrated with different doses of Omp22 and the possible acute toxicity was assessed. The results demonstrated that the posture, reaction, appetite, coat, eyes and excrement had no abnormal symptoms in all mice. The change of bodyweight was monitored and showed no apparent difference between the mice receiving Omp22 and normal mice (Fig. 6A). Further, histological analyses on main organs collected from mice receiving Omp22 administration, including heart, liver, lung, kidney, and brain, didn’t show any pathological changes (Fig. 6B). All the mice survived in this experiment.

## Discussion

Outer membrane proteins (OMPs) play important roles in bacterial survival, virulence^21^,^22^, cell adhesion^23^, host invasion^24^, and immune evasion^22^,^25^. OMPs are usually immunogenic and exposed on the cell surface, and appear to be potential antigen candidates. Some OMPs have been shown to associate with antimicrobial resistance^26^,^27^,^28^,^29^,^30^,^31^,^32^,^33^. They form channels and participate in the influx of antibiotics such as carbapenems, and the loss or reduced expression of these OMPs influences drug permeability^34^. Considering variations in structure, richness, and distribution of some OMPs as a survival mechanism to escape from antibacterial pressure have been developed by many bacteria, the potentials of targeting an outer membrane protein to elicit protective immunity need to be carefully investigated.

Previous studies showed some protein components of *A. baumannii* OMVs were highly immunogenic. And therefore, they were considered potential antigen candidates for further identification and assessment in our studies. On this basis, using data of reported clinical *A. baumannii* isolates, bioinformatics analyses on sequence conservation and possible classification/function of candidate proteins were performed. The highly conserved proteins and predicted membrane or secretion proteins were chosen preferentially. And more, the proteins that may be involved in antimicrobial resistance^26^,^27^,^28^,^29^,^30^,^31^,^32^,^33^,^34^, especially those with absent expression, were avoided to choose as an ideal vaccine target. At last, the candidate proteins were expressed in *E. coli* cells. Omp22 was one of those being efficiently folded into soluble form and purified with affinity chromatography of nickel-agarose. Since affinity purification requires the formation of Histidine-patch^35^, the results indicated that Omp22 might have properly folded and possessed nature immunogenic epitopes. Taken together, Omp22 was chosen and reported in the current study for the first time that it is a potent antigen candidate for developing an effective vaccine or preparing antisera to control *A. baumannii* infections. High conservation of Omp22 might provide effective protection against varied clinical *A. baumannii* strains. And, extremely low homology to human proteins implies that the risk for Omp22-elicited antibodies to cross-recognize human proteins is theoretically almost negligible. We detected Omp22 level in 14 clonally distinct clinical isolates with multiple-drug resistance, 13 of which showed a similar expression level except for Ab1 which was clearly lower than the others (Fig. 1D). Till now, there was no absence of Omp22 expression was reported in drug-resistant *A. baumannii.* In opsonophagocysis assays, the mean killing activities of antiserum was 39.8% or 36.5% on Ab1, 37.48% on Ab3, 47.66% on Ab4, 54.4% on Ab7, and 42.6% on Ab14 (Fig. 4C), which is basically at a similar level. The data demonstrated that Omp22 expression was basically stable in clinical isolates showing multiple-drug resistance and different drug resistant profiles; and, even if Omp22 was low expressed, antiserum was still able to mediate enough opsonophagocytic killing effects against *A. baunmanii* cells.

Although many features of human sepsis are not recapitulated, bacterial infection models have provided important insights into mechanisms of the host response to pathogens. Inoculation of animals with bacterial flora has been a common tool^36^,^37^,^38^. However, the transient introduction of high doses of bacteria to establish high bacteremia counts does not necessarily represent a reliable sepsis model, and as high doses as 10^11^ to 10^12^ CFU of bacteria commonly administered do not typically colonize and replicate within the host, often due to rapid lysis by complement^39^. This may lead to a potential model of intoxication with endotoxins rather than a true model of infection^39^,^40^. And thus, this model has limitations for sepsis study, especially for investigating the mechanism or developing a clinically applicable medicine. In contrast, models permiting an experimental host to be efficiently infected at relatively low doses and presenting an evolution of sepsis from a focus of infection more accurately reflect the course of human sepsis and facilitate the extrapolation of animal data to clinical efficacy in septic patients. However, this model depends on careful choosing of bacteria and/or host and is not easy to obtain. In some studies, neutropenia or diabetic mice have been used to make mice susceptible to lethal infection caused by *A. baumannii*, in which lower doses of bacteria (1.4–2 × 10^7^ CFU) were used and mice survived longer while infection was established. Those models allow the development of pulmonary inflammation^41^,^42^,^43^ or resembling a clinical risk factor of *A. baumannii* infection such as diabetes to some extend^17^. In the current study, a common protocol with a dose of 1 × 10^6^ CFU of bacteria mixed with 10% porcine mucin was used. Generally, the control mice died within 24 hour or 48 hour as shown in our and other groups’ similar studies^9^,^12^,^44^. The reason may attribute to that, invaded *A. baumannii* cells are quickly cleared in normal mice; only when the administrated bacterial load is high enough to allow survival bacteria to release toxic material such as endotoxin and produce serious damage will the death of experimental animals occur. Considering *A. baumannii* is a conditioned pathogen and doesn’t invade into host cells in general, the most possible effective mechanism of a vaccine is producing specific antibodies to promote opsono-phagocytosis and thus reduce the survival and proliferation of infected bacteria and the release of harmful mediators, which is not dependent on directly targeting the immune pathogenesis of sepsis. From this perspective, the model is appropriate for antigen identification and vaccine assessment. Both active and passive immunization strategies effectively protected mice from lethal challenge of Ab1 strain, evidenced by increased survival of mice, suppressed bacterial burden and reduced accumulation of proinflammatory cytokines in immunized mice. As mentioned above, Ab1 strain presented a significantly low richness of Omp22 as compared to the other 13 isolates, which didn’t produce significant influences on eliciting effective immune protection *in vivo*. Further, we investigated the possible mechanism underlying specific *in vivo* clearance of infected bacteria after Omp22 immunization. Through *in vitro* opsonophagocysis assays with endogenous complement components inactivated or with phagocytes removed, we demonstrated that specific antibody/complements-mediated opsonophagocytic killing effects was probably responsible for the *in vivo* bactericidal effects. On this basis, we believed that the richer the target protein distribution on bacterial cells is, the more chances bacteria hold to be bound by specific antibodies and then cleared by immune system. And thus, we chose Ab1 which has a lower expression of Omp22 than the other isolates for assessing vaccine effectiveness in sepsis model, and expected that the isolates with higher Omp22 expression should also be effectively protected. To some extent, it is supported by the *in vitro* opsonophgocytosis assay that the killing activities mediated by Omp22 antisera on the other Ab isolates were at least not lower than that on Ab1. Furthermore, in the sepsis model passive immunization with Omp22 antisera effectively protected mice from lethal challenge of Ab4 and Ab14 which have higher Omp22 expression than Ab1.

Both a tumor cell line and a normal cell line were used in the study to roughly test *in vitro* the possible toxicity of Omp22. The results that the cells incubated with as high as 80 μg/ml of Omp22 remained above 80% viability indicated that basically Omp22 is harmless and only a high concentration of the protein preparation presented mild suppressing effects on cell growth. And, it can not be excluded that the contaminants in the protein preparation exerted the effects. Previous studies reported that 20 μg/ml *A. baumannii* OmpA caused a 10% reduction in the conversion of MTT to formazan crystals in incubated A549 cells, and 40 and 80 μg/ml of OmpA produced a greater than 75% reduction^20^. Further, a concentration of 3 μg/ml of OmpA resulted in approximately 50% cell death and 6 μg/ml produced approximately 100% cell death in human laryngeal epidermoid carcinoma Hep-2 cells. The reported results also suggested that it was likely that using different cell lines could affect the concentration of OmpA necessary for inducing cell death^45^, whereas we displayed that Omp22 preparation only showed mild suppressive effects on cell growth in both A549 and 293FT cell lines.

The biological functions of Omp22 remain unclear till now. Bioinformatics analyses were conducted to suggest its protein classification and possible functions. Domain analysis revealed Omp22 might be an “outer membrane protein-related peptidoglycan-associated (lipo) protein”, with a glycine zipper on the position of 37–81 and a peptidoglycan (PG) binding domains similar to the C-terminal domain of OmpA (OmpA_C-like) on the position of 110–213 (supplementary Figure 1A). *In silica* homology model of Omp22 was built, and it displayed that Omp22 did not fold into a classic porin-like protein structure^46^ (supplementary Figure 1B). As known, porin is tightly involved into drug-resistance mechanism of bacteria and easy variations in expression and structure of porin prevent it to be a promising vaccine candidate. Omp22 has an OmpA_C-like domain, and might interact with PG^47^, indicting that Omp22 might modulate the biogenesis of *A. baumannii* OMVs, because linkages between OMP and PG are directly responsible for OMVs production (supplementary Figure 1C)^48^,^49^. As shown, specific antibodies-mediated opsonophagocytosis or activated classic complement pathway may be common mechanisms of vaccination with Omp22 or the other outer membrane proteins. Since the function of Omp22 has not been identified yet, it’s unclear whether Omp22 vaccination performs through distinct mechanism from the other proteins. Considering *A. baumannii* is a conditioned pathogen and in general it does not invade host cells, cellular immune or blocking interaction between bacteria and host cells by neutralizing antibodies will not be considered in this case. However, it should not be excluded that specific antibodies induced by a vaccine take effects by blocking virulence factor.

It is not clear if using a single protein will be sufficient in clinics. *A. baumannii* IWC^9^, OMVs^11^,^12^ and OMC^10^ have been shown to be extremely effective in animals, due to included multiple antigenic components and immune stimulators. OMCs may have better safety than the other two, however, it still has many useless and even harmful components for eliciting specific protective immune in human. It’s quite promising to identify effective and safe antigen candidates and their combination to replace the applications of OMCs. Worth to be noted, using gene recombinant technology, an interested protein can be prepared in quantity to booster specifically its protective immune responses, while it might present only in a low richness in nature OMCs preparation and thus fail to induces effective immune response. It has been proven that a single protein, such as OmpA, Bap, and Ata, is effective in preventing *A. baumannii* infection in animal models. However, there may be limitations for a single protein to be successfully applied in clinics. The immunogenicity of a subunit protein might be weak in human when administered alone and thus require often to be combined with an adjuvant^50^, conjugated to polysaccharide or protein carriers^51^,^52^, or formulated in controlled-release systems. A single protein may fail to provide sufficient protective effects against pathogen infection due to the limitation of included effective antigenic epitopes. The expression level and even amino acids sequence might change in varied clinical strains, and thus targeting to a single protein may have limited broadly protective effects and combination of homologous proteins from varied strains is required. Taken together, it’s likely that combining several proteins is required for constituting a clinically effective vaccine. For example, a four component meningococcal B vaccine (Bexsero^®^) consists of three recombinant proteins (NadA, fHbp, NHBA) and OMVs have been clinically used in some countries^53^. And more recently, the lipoprotein factor H-binding protein (fHbp)^54^,^55^ -based MenB vaccine has been approved in the USA (rLP2086, Trumenba^®^), indicating even a single protein can be an effective vaccine in clinics.

Overall, in this study, Omp22 was demonstrated to be highly immunogenic, conserved, of immune protection, and probably safe in human, and thus is a promising antigen candidate to be used for developing an effective vaccine or preparing antiserum to provide an optional approach in combating *A. baumannii* infection.

## Materials and Methods

### Ethics Statement

The animal experimental procedures were approved by the Ethics Committee of Animal Care and Welfare, Institute of Medical Biology, Chinese Academy of Medical Sciences (CAMS) & Peking Union Medical College (PUMC) (Kunming, China) (Permit Number: SYXK (dian) 2010-0007), in accordance with the animal ethics guidelines of the Chinese National Health and Medical Research Council (NHMRC). All efforts were made to minimize animal suffering.

### Mice and *A. baumannii* strains

Female ICR mice (6–8 weeks of age) were raised and maintained in the Central Animal Care Services of the institute, under specific pathogen-free (SPF) conditions. The *A. baumannii* ATCC 17978 strain was obtained from American Type Culture Collection (ATCC). Totally 14 clinical *A. baumannii* strains were collected from the intensive care units (ICUs) of two different hospitals. Strains 1 to 4 (Ab1 to Ab4) were isolated from the First Affiliated Hospital of Kunming Medical College (Kunming, China), and strains 5 to 14 (Ab5 to Ab14) were collected from the Second Affiliated Hospital. All clinical *A. baumannii* strains were confirmed to be multi-drug resistant (MDR) strains by drug sensitivity experiments.

### Gene amplification and amino acid sequence analysis of Omp22

The inoculations of Ab1-14 were grown in Luria-Bertani (LB) medium under the dual selection pressures of antibiotics ampicillin and kanamycin. Collected cells were used for the amplification of Omp22 nucleotide sequences by PCR. The primers were Omp22-F (5′ GGATCC ATG CGT GCA TTA GTT ATT T 3′) and Omp22-R (5′ GAATTC TTA TTG TTT AGC ATA AAT G 3′), which designed according to the Omp22 sequence of *A. baumannii* ATCC 17978 (Omp22: Assession number YP_001083918). The PCR products were cloned into the pMD19-T simple vector (Takara) and used for DNA sequencing. The sequencing results were analyzed using the DNASTAR.Lasergene.v7.1 software (USA). Moreover, the amino acid sequence conservation and amino acid domain similarity to reported *A. baumannii* strains, and amino acid homology with human proteins were analyzed using NCBI BLAST. Omp22 was modeled with homology in silica using the SWISS-MODEL automated protein structure homology modeling server (available at http://swissmodel.expasy.org)^56^,^57^,^58^,^59^.

### Expression and purification of Omp22

Gene encoding for Omp22 was obtained by PCR amplification, using the genomic DNA of *A. baumannii* ATCC 17978 strain as the template. The primers were Omp22-F and Omp22-R. The PCR product was digested using the *Bam*HI and *Eco*RI enzymes (Takara) and ligated into the plasmid pThioHisA (Invitrogen). The recombinant plasmid pThioHisA-Omp22 was transformed into *E. coli* BL21 (DE3). The recombinant protein thioredoxin (Trx)-Omp22 was induced with isopropyl-β-d-thiogalactoside (IPTG) (Sigma) at 37 °C for 4 h. The inclusion bodies were washed with wash buffer (1 M urea in binding buffer (20 mM Tris-HCl, pH 8.8)) and dissolved in solution buffer (6 M urea in binding buffer). The dissolved inclusion bodies were dialyzed with binding buffer at 4 °C overnight for refolding, and then purified with affinity chromatography of HisTrap FF column (GE Healthcare). After eluted with elution buffer (1 M imidazole in binding buffer), the collected fractions were analyzed with SDS-PAGE.

### Cytotoxic activity of Omp22 *in vitro*

A cytotoxicity assay was performed following the manufacturer’s instructions for the CellTiter 96^®^ AQueous One Solution Cell Proliferation Assay (Promega)^60^. Briefly, A549 cells (a human lung epithelial cell line) and 293FT cells (human embryonic kidney cells) were maintained in DMEM supplemented with 10% FBS, 100 U penicillin/ml, and 100 μg streptomycin/ml at 37 °C with 5% CO_2_. For the cell viability assays, solutions containing 0, 20, 40, or 80 μg/ml of purified Trx-Omp22 were prepared in the medium. The concentrations of both the A549 and 293FT cells were adjusted to ~1 × 10^5^ cells/ml (100 μl/well), incubated at 37 °C for 24 h, and then 20 μl of MTS reagent was added to each well. After an additional incubation for 2 h at 37 °C, the optical density at 490 nm (OD_490_) was determined for each well. The proliferation rate is calculated by comparing the OD_490_ values for Omp22-stimulated cells and medium only-treated cells.

### Mouse immunizations and sepsis models

Mice were immunized subcutaneously (s.c.) with different doses of Trx-Omp22 proteins at 5, 10, 20, or 50 μg with 1 mg Alum (Thermo scientific) as adjuvant, on days 0, 14, and 28. Blood samples were collected at days 21, 35, and 49. To study active immunization, mice were immunized with 50 μg Omp22, and then 1 × 10^6^ CFU/200 μl of clinically isolated Ab1 cells with 10% porcine mucin (w/v; Sigma-Aldrich) was administered intraperitoneally (i.p.) to each mouse 3 weeks after the last immunization^12^. For passive immunization, 100 μl of antisera collected from immunized mice was injected intravenously (i.v.) into the mice 1 hour before challenging with *A. baumannii* Ab1 (1 × 10^6^ CFU), Ab4 (2.1 × 10^6^ CFU) or Ab14 (1.6 × 10^6^ CFU) strains; the sera were collected from mice that received only the adjuvant served as the control. The bacterial burdens in the blood and major organs (lung, spleen, liver, and kidney) of the mice were measured 12 h after challenging with *A. baumannii*. The levels of inflammatory cytokines in the serum were detected. The mice were monitored continuously for seven days to determine the survival rate. Specific IgG responses in serum were measured with enzyme-linked immunosorbent assays (ELISAs). Briefly, OMVs prepared from *A. baumannii* ATCC 17978 were used to coat microplates at 5 μg of total protein per 100 μl in each well. Plates were then incubated with the serum samples, incubated with anti-mouse IgG secondary antibodies (diluted 1:10,000, Santacruz), and developed with alkaline phosphatase substrate. The endpoint titer was defined as the highest dilution at which the optical density at 405 nm was at least 0.1 above that of the background wells; in the background wells, serum samples were replaced by PBS^61^. The concentrations of cytokines (IL-1β, IL-6, TNF-α, IFN-γ, and MCP-1) in serum were detected using ELISA, which was performed using paired capture and biotinylated detection antibodies purchased from eBioscience, Inc. (USA) according to the supplier’s instructions. For the immune blotting assays, equal amounts of whole cell proteins (WCs) from the clinical isolates, which normalized by OD_600_ value, were separated on 12% SDS-PAGE gels, and then transferred onto a PVDF membrane. The membrane was incubated with a pooled antiserum sample at a dilution of 1:1000 for 1 h, and then incubated with a horseradish peroxidase-coupled anti-mouse IgG secondary antibody (Invitrogen) at a 1:10,000 dilution. The PVDF-membrane was developed with ECL (Thermo Scientific) and used to expose X-ray film (Kodak).

### Complement and opsonophagocysis assays

Murine macrophage RAW264.7 cells were cultured in DMEM/HIGH glucose (HyClone) with 10% fetal bovine serum (FBS). The cells were activated with a 3-day exposure to 100 nM phorbol 12-myristate 13-acetate (PMA; Sigma-Aldrich). Harvesting was performed by scraping with cell scrapers (Thermo Scientific). The macrophages (~2 × 10^6^ cells/ml, 80 μl/well) and the clinical isolates Ab3, Ab4, Ab7, and Ab14 (~7 × 10^5^ CFU/ml, 10 μl/well) were added separately to the microwells. The pooled Trx-Omp22 antiserum, adjuvant control serum and naïve serum were serially diluted to final concentrations of 1:10, 1:100 and 1:1000 with culture medium (10 μl/well). A subset of sera was subjected to heat treatment at 56 °C for 30 min to inactivate the endogenous complement components. The test sera, bacteria, and macrophages were mixed and incubated for 1 h with gentle shaking. Finally, the mixtures were diluted and plated for bacterial counting. Serum killing rates are calculated by comparing the number of the reduced CFU with naïve serum.

### Acute toxicity of Omp22 *in vivo*

Acute toxicity test of Omp22 was performed following good laboratory practice (GLP) guidelines using male and female ICR mice. Eight week-old mice (n = 4/group) were given a subcutaneous injection with PBS or Omp22 of 5, 50, or 500 μg per mouse, respectively. Mice were monitored at intervals of 2 day for changes in “clinical” signs such as appearance, behavior, activity and body weight. At day 14, mice were euthanized, and then a necropsy was performed. The brain, liver, spleen, heart, lung and kidney were harvested and stained with hematoxylin and eosin (H&E).

### Statistical analyses

All statistical analyses were performed using GraphPad Prism 5.0 (GraphPad Software, Inc.,). Survival rates were compared using the non-parametric log-rank test. The bacterial burdens, cytokine levels were analyzed with an unpaired Student’s *t* test. One-way ANOVA with Tukey’s multiple comparison test was applied to analyze cytotoxic activity and opsonic activity. Differences were considered significant if the *p* value was < 0.05. (*P < 0.05; **P < 0.01; ***P < 0.001). All graphed values represent the mean, and the error bars represent the standard error.

## Additional Information

**How to cite this article**: Huang, W. *et al.* Immunization with a 22-kDa outer membrane protein elicits protective immunity to multidrug-resistant *Acinetobacter baumannii.* *Sci. Rep.* **6**, 20724; doi: 10.1038/srep20724 (2016).

## Supplementary Material

Supplementary Information
