# Supplementary material for: Immunization with a 22-kDa outer membrane protein elicits protective immunity to multidrug-resistant Acinetobacter baumannii
Source: Sci Rep. 2016 Feb 8;6:20724. doi: 10.1038/srep20724 (PMC4745112; doi:10.1038/srep20724)

Immunization with a 22-kDa outer membrane protein elicits protective immunity to multidrug-resistant *Acinetobacter baumannii*

Weiwei Huang[1,2,3], Yufeng Yao[1,2,3], Shijie Wang[1,2,3], Ye Xia[1,2,3], Xu Yang[1,2,3], Qiong Long[1,2,3], Wenjia Sun[1,2,3], Cunbao Liu[1,2,3], Yang Li[1,2,3], Xiaojie Chu[1,2,3], Hongmei Bai[1,2,3], Yueting Yao[1,2,3], Yanbing Ma[1,2,3*]

1 Laboratory of Molecular Immunology, Institute of Medical Biology, Chinese Academy of Medical Sciences & Peking Union Medical College; Kunming, China 650118,

2 Yunnan Key Laboratory of Vaccine Research & Development on Severe Infectious Diseases; Kunming, China 650118,

3 Yunnan Engineering Research Center of Vaccine Research and Development on Severe Infectious Diseases, Kunming, China 650118

***Corresponding:**

Prof. Yanbing Ma

Laboratory of Molecular Immunology,

Institute of Medical Biology,

Chinese Academy of Medical Sciences & Peking Union Medical College,

Kunming, China 650118

Email: yanbingma1969@126.com

Tel: +86 871 68339287

Supplementary figure legends

Supplementary figure 1. Bioinformatics analysis of Omp22

(A) Amino acid domain analyses on Omp22. The picture showed that a glycine zipper on the position of 37-81 (blue), and a peptidoglycan (PG) binding domains similar to the C-terminal domain of outer-membrane protein OmpA (OmpA_C-like) (green) on the position of 110-213. (B) Omp22 was homology modeled *in silica* by using the SWISS-MODEL automated protein structure homology modeling server. (C) Bacterial outer membrane vesicles (OMVs) production model. Overview of Gram-negative envelope architecture in the context of OMVs production. “Free” or “bound” forms of lipoprotein (red), porin (green) and outer membrane proteins (yellow) have been showed in picture.

Supplementary Figure 1

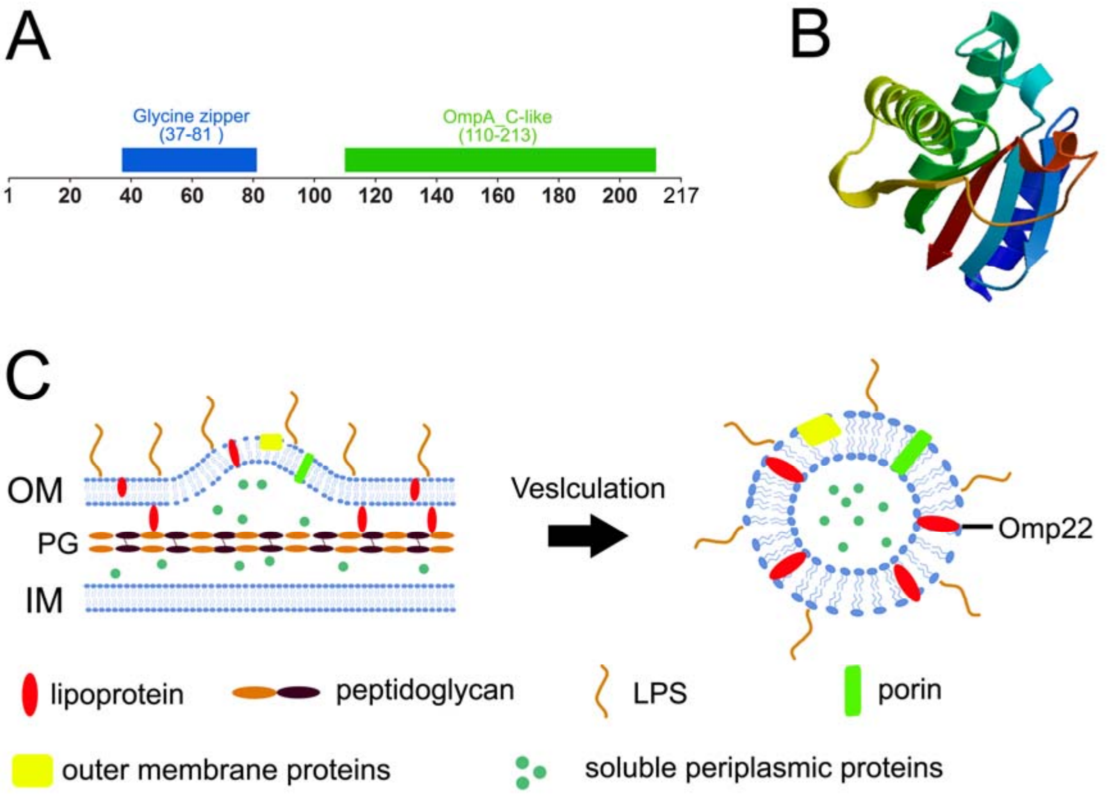

Supplement: Supplementary Information [file srep20724-s1.pdf]
